# Direct oral anticoagulants compared to low‐molecular‐weight heparin for the treatment of cancer‐associated thrombosis: Updated systematic review and meta‐analysis of randomized controlled trials

**DOI:** 10.1002/rth2.12359

**Published:** 2020-05-21

**Authors:** Florian Moik, Florian Posch, Christoph Zielinski, Ingrid Pabinger, Cihan Ay

**Affiliations:** ^1^ Clinical Division of Haematology and Haemostaseology Department of Medicine I Comprehensive Cancer Center Vienna Medical University of Vienna Vienna Austria; ^2^ Division of Oncology Department of Internal Medicine Comprehensive Cancer Center Graz Medical University of Graz Graz Austria; ^3^ Vienna Cancer Center Vienna Hospital Association and Medical University Vienna Vienna Austria

**Keywords:** anticoagulants, factor Xa inhibitors, low molecular weight heparin, neoplasms, venous thromboembolism, venous thrombosis

## Abstract

**Background:**

Low‐molecular‐weight‐heparins (LMWHs) have been established for the treatment of cancer‐associated venous thromboembolism (VTE). Recently published randomized controlled trials (RCTs) have compared direct oral anticoagulants (DOACs) with LMWHs. The aim of this systematic review and meta‐analysis was to evaluate efficacy and safety of DOACs versus LMWHs and update the evidence for treatment of VTE in cancer.

**Methods:**

Biomedical databases were screened for RCTs evaluating DOACs for cancer‐associated VTE. Primary efficacy and safety outcomes of this meta‐analysis were recurrent VTE and major bleeding at 6 months. Secondary outcomes comprised clinically relevant nonmajor bleeding (CRNMB), major gastrointestinal (GI) and genitourinary bleeding, mortality, fatal bleeding/pulmonary embolism, and treatment discontinuation rate. We performed prespecified subgroup analyses. Pooled relative risk (RR) and 95% confidence intervals (CIs) were obtained by the Mantel‐Haenszel method within a random‐effect model.

**Results:**

We screened 759 articles and included 4 RCTs (n = 2894). DOACs significantly reduced recurrent VTEs compared to LMWHs (5.2% vs 8.2%; RR, 0.62 [95% CI, 0.43‐0.91]), but were associated with a nonsignificant increase in major bleedings (4.3% vs 3.3%; RR, 1.31 [95% CI, 0.83‐2.08]) and a significant increase in CRNMB (10.4% vs 6.4%; RR, 1.65 [95% CI, 1.19‐2.28]). Mortality risks were comparable between groups (RR, 0.99 [95% CI, 0.83‐1.18]). Preterm treatment discontinuation was less common with DOACs (RR, 0.88 [95% CI, 0.81‐0.96]). Major bleeding was more frequent in patients with GI cancer treated with DOACs (RR, 2.30 [95% CI, 1.08‐4.88]).

**Conclusion:**

In patients with cancer‐associated VTE, DOACs are more effective in preventing recurrent VTE compared to LMWH. However, risk of bleeding is increased with DOACs, especially in patients with GI cancer.

**Essentials**

- Recent randomized controlled trials compared DOACs to LMWHs for cancer‐associated VTE.
- A meta‐analysis of aggregated safety and efficacy outcomes of DOACs versus LMWHs was conducted.
- Recurrent VTE was less frequent with DOACs, but risk of bleeding was increased.
- Patients with gastrointestinal cancer had more major bleedings with DOACs than with LMWHs.

## 1 INTRODUCTION

Patients with cancer are at an increased risk of developing venous thromboembolism (VTE), which is a major contributor to morbidity and mortality.^1^, ^2^, ^3^, ^4^ As compared to VTE in the noncancer setting, managing cancer‐associated VTE is challenged by a higher risk of recurrence and increased risk of major bleeding during anticoagulant treatment.^5^ The Comparison of Low‐Molecular‐Weight Heparin Versus Oral Anticoagulant Therapy for the Prevention of Recurrent Venous Thromboembolism in CLOT (Patients With Cancer ) study and subsequent trials have tested the efficacy and safety of low‐molecular‐weight heparins (LMWHs) versus vitamin K antagonists (VKAs) for the treatment of VTE in patients with cancer, with favorable results for LMWH (ie, reduced risk of recurrence and no increase in risk of bleeding).^6^, ^7^, ^8^ Based on these studies, guidelines have uniformly endorsed LMWH monotherapy as the standard‐of‐care treatment of VTE in cancer‐associated VTE for 3‐6 months until recently.^3^, ^7^

Direct oral anticoagulants (DOACs), such as apixaban, edoxaban, rivaroxaban, and dabigatran, have emerged as the preferred treatment option for VTE in the general population.^9^, ^10^, ^11^ However, the subgroup of patients with cancer included in trials testing DOACs for VTE was limited, and the control treatment in these trials was VKA. As the preferred treatment for cancer‐associated VTE at that time was LMWH, no robust data for efficacy and safety of DOACs for patients with cancer‐associated VTE were available until recently. Therefore, no definitive conclusion could be drawn for the use of DOACs in patients with active cancer and a direct comparison of DOACs to LMWHs was urgently needed.^5^

Recently, DOACs have been tested for the treatment and secondary prevention of VTE in patients with cancer head‐to‐head against LMWHs according to the CLOT regimen (dalteparin 200 IU/kg for 1 month, followed by dalteparin 150 IE/kg) in 4 studies, which provide evidence for the efficacy and safety of DOACs, in particular factor Xa inhibitors (apixaban, edoxaban, and rivaroxaban).^12^, ^13^, ^14^, ^15^ DOACs have been shown to be at least noninferior compared to LMWH monotherapy for the treatment of cancer‐associated VTE. Relevant safety outcomes such as rates of bleeding events differed in these studies. Further, these trials also included patients with incidentally diagnosed asymptomatic VTE, which is frequently observed in patients with cancer.

Previous meta‐analyses have been performed comparing DOACs to LMWHs for the treatment of cancer‐associated VTE aggregating data from 2 or 3 of the now 4 available randomized controlled trials (RCTs) and showed a nonsignificant decrease in risk of VTE accompanied by an increase in risk of bleeding in patients treated with a DOAC.^16^, ^17^, ^18^, ^19^

The aim of this systematic review and updated meta‐analysis was to compare efficacy and safety of DOACs versus LMWHs for the treatment of acute cancer‐associated VTE by aggregating results from all available RCTs and to assess their relative benefit in specific subgroups.

## 2 METHODS

We conducted a systematic review of the literature and meta‐analysis to identified RCTs comparing DOACs with LMWHs specifically in patients with cancer. The study was conducted in accordance with the *Cochrane Handbook for Systematic Reviews of Interventions*.^20^ The proposal of the systematic review, including strategy of literature research, was submitted online to the International Prospective Register of Systematic Reviews prior to the initiation of literature review and identification of eligible studies.

### 2.1 Literature research and study selection

Two researchers (FM and CA) independently conducted a review of the literature (April 1, 2020), using the biomedical databases EMBASE, MEDLINE, and CENTRAL. Predefined search terms were used combined with filters to identify clinical trials (complete search strategy is provided in Appendix S1). Titles and abstracts of primarily identified publications matching search criteria were screened for conformity with inclusion and exclusion criteria. The remainder of studies underwent full‐text evaluation for eligibility.

To be eligible for inclusion, studies had to fulfil all predefined inclusion criteria and not match any exclusion criteria. Criteria for inclusion were defined as follows: (i) prospective clinical trials testing DOACs specifically for the treatment of cancer‐associated VTE, (ii) control arm of patients treated with LMWHs, (iii) randomized study group allocation, (iv) adult patients only (≥18 years of age), and (v) qualifying VTE event must include symptomatic or asymptomatic/incidental pulmonary embolism (PE) or deep vein thrombosis (DVT). Exclusion criteria were (i) observational cohort study, (ii) lack of control group, (iii) no randomization process for study group allocation, and (iv) inclusion of patients <18 years of age.

Any disagreements between the 2 reviewers were resolved by discussion, involving also the co‐authors.

### 2.2 Data extraction and study outcomes

Identified studies that were found eligible for inclusion underwent data extraction. Baseline information of our selected studies including characteristics on study design, and respective inclusion and exclusion criteria were collected. Baseline characteristics of individual patient cohorts including age, sex, stage and type of cancer, and specifics regarding qualifying VTE diagnosis were gathered.

Extracted outcome data comprised efficacy and safety results of included studies. The primary efficacy outcome of the meta‐analysis was defined as the aggregated rate of recurrent VTE at 6 months, and the primary safety outcome was defined as the 6‐month rate of major bleeding. Secondary outcomes included rates of clinically relevant nonmajor bleeding (CRNMB), any bleeding, major gastrointestinal (GI) bleeding, major genitourinary (GU) bleeding, intracranial bleeding, mortality, fatal bleeding, PE‐related mortality, and rate of preterm discontinuation of anticoagulation. All extracted outcome variables were defined as within the respective study. For calculating aggregated rates of outcomes, we used modified intention to treat populations of the selected studies. Prespecified subgroup analyses were conducted in patients with GI cancer and incidental VTE as index event.

Risk of bias of included studies was evaluated with the modified Cochrane risk‐of‐bias tool.^21^ Risk of publication bias was assessed within a funnel plot for the primary efficacy outcome.

### 2.3 Statistical analysis

Aggregated summary statistics were calculated by weighted means and proportions, as suitable according to type of variable. Pooled relative risk (RR) and corresponding 95% confidence intervals (CIs) of outcome variables were obtained by combining results from selected studies by the Mantel‐Haenszel method within a random‐effects model. Assessment of heterogeneity was conducted graphically from forest plots and tested by assessing the I^2^ as a measure of variation in RR attributable to heterogeneity among included studies. In the case of a substantial degree of heterogeneity between studies, outcome analysis was repeated after exclusion of selected studies.

All analyses were performed with Stata (Windows version 15.0, Stata Corp., Houston, TX, USA).

## 3 RESULTS

### 3.1 Study identification

**FIGURE 1:**
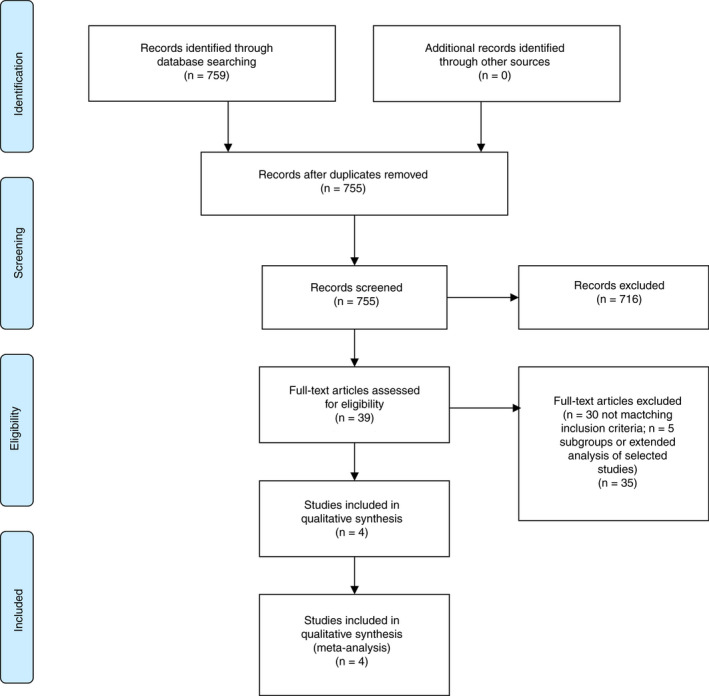
Preferred Reporting Items for Systematic Reviews and Meta‐Analyses (PRISMA) flow diagram for study selection

A total of 759 published records, matching the predefined search terms, were found by systematic database search (EMBASE, 396; CENTRAL, 147; MEDLINE, 216). After removal of duplicates, 755 records were screened for eligibility. After exclusion of 716 records based on information drawn from title and abstract, 39 records were selected for full‐text assessment. Of those, we identified 4 clinical trials contributing to 9 different publications, including 5 publications on specific subgroups or post hoc analysis or on outcomes during extended treatment beyond 6 months.^12^, ^13^, ^14^, ^15^, ^22^, ^23^, ^24^, ^25^, ^26^ Figure 1 shows a Preferred Reporting Items for Systematic Reviews and Meta‐Analyses flow diagram, which summarizes the process of study identification and selection.^27^

### 3.2 Study characteristics

Selected studies found eligible for inclusion were the Hokusai VTE Cancer trial, SELECT‐D (Anticoagulation Therapy in Selected Cancer Patients at Risk of Recurrence of Venous Thromboembolism), ADAM VTE (Apixaban and Dalteparin in Active Malignancy Associated Venous Thromboembolism) trial, and the Caravaggio (Apixaban for the Treatment of Venous Thromboembolism in Patients With Cancer) study.^12^, ^13^, ^14^, ^15^ These studies all represent RCTs, which compared DOACs with LMWHs for the treatment of VTE in patients with cancer. Specifics on study design and corresponding inclusion and exclusion criteria are presented in Table 1 and the rates of outcome variables within selected studies in Table 2.

**TABLE 1:** Study design and baseline characteristics of selected trials

Trial name	Hokusai VTE Cancer	SELECT‐D	ADAM VTE	Caravaggio
First author and year of publication	Raskob, 2018	Young, 2018	McBane, 2019	Agnelli, 2020
Study design	Randomized open‐label noninferiority trial (blinded adjudication of outcomes)	Randomized, open‐label, multicenter pilot trial (blinded adjudication of outcomes)	Investigator initiated phase IV, multicenter, randomized, open‐label, superiority trial	Randomized, controlled, investigator‐initiated, open‐label, noninferiority trial (blinded adjudication of outcomes)
No. of patients	1046	406	300	1155
Intervention (DOAC)	Edoxaban (60 mg 1×/d after LMWH for 5 d)^a^	Rivaroxaban (15 mg 2×/d 3 wk → 20 mg 1×/d)	Apixaban (10 mg 2×/d 7 d → 5 mg 2×/d)	Apixaban (10 mg 2×/d 7 d → 5 mg 2×/d)
Comparator (LMWH)	Dalteparin (200 IU/kg 1×/d 1 mo → 150 IU/kg 1×/d)	Dalteparin (200 IU/kg 1×/d 1 mo → 150 IU/kg 1×/d)	Dalteparin (200 IU/kg 1×/d 1 mo → 150 IU/kg 1×/d)	Dalteparin (200 IU/kg 1×/d 1 mo → 150 IU/kg 1×/d)
Treatment duration	12 mo^b^	6 mo	6 mo	6 mo
Study participants	Adult pts. with cancer (active or diagnosed within 2 y)^c^ + acute VTE	Adult pts. with active cancer^c^ + acute VTE	Adult pts. with active cancer^d^ + acute VTE	Adult pts. with cancer (active or diagnosed within 2 y)^c^
Qualifying type of acute VTE	– Symptomatic/incidental proximal DVT – Symptomatic PE ‐ incidentally detected PE of segmental or more proximal pulmonary arteries	– Symptomatic lower extremity proximal DVT – symptomatic/incidental PE	– Lower and upper extremity DVT – PE – Splanchnic or cerebral vein thrombosis	– Symptomatic/incidental proximal – Lower extremity DVT – PE
Excluded cancer populations (selection)	– Basal cell/squamous cell cancer of the skin – ECOG 3‐4	– Basal cell‐/Squamous cell cancer of the skin – ECOG 3‐4 – Weight ≤ 40 kg	– Basal cell‐/Squamous cell cancer of the skin – ECOG 3‐4	– Basal cell‐/Squamous cell cancer of the skin – Cerebral metastasis, primary CNS tumors, acute leukemia – ECOG 3‐4
Active cancer, n (%)	1024 (97.9)	406 (100)	300 (100)	1124 (97.3)
Age	Mean: 64.0 y	Median: 67	Mean: 62.7	Mean: 67.2
Male sex, n (%)	540 (51.6)	214 (52.7)	145 (48.3)	568 (49.2)
ECOG 0, n (%)	303 (29.6)	119 (29.3)	122 (40.7)	356 (30.8)
ECOG 1, n (%)	489 (46.7)	185 (45.6)	146 (28.7)	558 (48.3)
ECOG 2, n (%)	247 (23.6)	95 (23.4)	32 (10.7)	241 (12.2)
Metastatic cancer, n (%)	554 (53.0)	236 (58.1)	193 (64.3)	785 (68.0)^e^
GI cancer, n (%)	305 (29.2)	177 (43.6)	105 (35.0)	375 (32.5)
Hematologic malignancy, n (%)	111 (10.6)	10 (2.5%)	28 (9.3)	85 (7.4)
Incidental VTE as index event, n (%)	340 (32.5)	113 (27.8)	NR	230 (19.9)
Primary outcome	Composite: recurrent VTE (symptomatic/incidental proximal DVT; symptomatic/incidental segmental or more proximal/fatal PE) + major bleeding	Recurrent VTE (proximal DVT; symptomatic/incidental/fatal PE; other sites: eg, subclavian vein, hepatic vein, inferior vena cava)	Major bleeding (according to ISTH definition^f^)	Recurrent VTE (proximal symptomatic/incidental DVT of the lower limbs; symptomatic DVT of the upper limbs; symptomatic/incidental segmental or more proximal/fatal PE)

Abbreviations: CNS, central nervous system; DOAC, direct oral anticoagulant; DVT, deep vein thrombosis; ECOG, Eastern Cooperative Oncology Group performance index; IU/kg, international units per kilogram of bodyweight; LMWH, low‐molecular‐weight heparin; NR, not reported; PE, pulmonary embolism; VTE, venous thromboembolism.

^a^ Or 30 mg 1×/d, if (i) body weight <60 kg, (ii) creatinine clearance of 30‐50 mL/min, or (iii) concomitant therapy with a potent P‐glycoprotein inhibitor.

^b^ Outcome variables during 6 mo of treatment were used for aggregating data within the meta‐analysis.

^c^ Active cancer defined as diagnosed within 6 mo, treatment within 6 mo, recurrent/metastatic cancer, hematologic cancer not in complete remission.

^d^ Active cancer defined as any evidence of cancer on cross‐sectional or positron emission tomography imaging, metastatic disease, and/or cancer‐related surgery, chemotherapy, or radiation therapy within the prior 6 mo.

^e^ Number of patients with metastatic disease not reported; number comprises patients with metastatic and locally advanced disease.

^f^ Defined as overt bleeding + (i) hemoglobin drop of ≥2 g/dL, (ii) transfusion of ≥2 units of packed red blood cells, (iii) bleeding in a critical site (intracranial, intraspinal/epidural, intraocular, retroperitoneal, pericardial, intraarticular, intramuscular with compartment syndrome), or (iv) fatal bleeding.

**TABLE 2:** Safety and efficacy outcomes in selected trials

Trial name	Hokusai VTE Cancer	SELECT‐D	ADAM VTE	Caravaggio
First author and year of publication	Raskob, 2018	Young, 2018	McBane, 2019	Agnelli, 2020
No. of patients: DOAC versus LMWH^a^	1046 (522 vs 524)	406 (203 vs 203)	287 (145 vs 142)	1155 (576 vs 579)
Recurrent VTE (DOAC vs LMWH)	7.9% vs 11.3% (41/522 vs 59/524) HR, 0.71 [0.48‐1.06]	3.9% vs 8.9% (8/203 vs 18/203) HR, 0.43 [0.19‐0.99]	0.7% vs 6.3% (1/145 vs 9/142) HR, 0.099 [0.013‐0.780]	5.6% vs 7.9% (32/576 vs 46/579) HR, 0.63 [0.37‐1.07]
Major bleeding (DOAC vs LMWH)	6.9% vs 4.0% (36/522 vs 21/524) HR, 1.77 [1.03‐3.04]	5.4% vs 3.0% (11/203 vs 6/203) HR, 1.83 [0.68‐4.96]	0.0% vs 1.4% (0/145 vs 2/142) HR, not estimable	3.8% vs 4.0% 22/576 vs 23/579 HR, 0.82 [0.40‐1.69]
CRNMB (DOAC vs LMWH)	14.6% vs 11.1% (76/522 vs 58/524) HR, 1.38 [0.98‐1.94]	12.3% vs 3.4% (25/203 vs 7/203) HR, 3.76 [1.63‐8.69]	6.2% vs 6.3% (9/145 vs 9/142) HR, 0.931 [0.43‐2.02]	9.0% vs 6.0% 52/576 vs 35/579 HR, 1.42 [0.88‐2.30]
Mortality (DOAC vs LMWH)	39.5% vs 36.6% 206/522 vs 192/524 HR, 1.12 [0.92‐1.37]	23.6% vs 27.6% 48/203 vs 56/203 nr	16% vs 11% (23/145 vs 15/142) HR, 1.40 [0.82‐2.43]	23.4% vs 26.4% 135/576 vs 153/579 HR, 0.82 [0.62‐1.09]
Major GI bleeding (DOAC vs LMWH)	3.8% vs 1.1% 20/522 vs 6/524	3.4% vs 2.0% 7/203 vs 4/203	0% vs 0% 0/145 vs 0/142	1.9% vs 1.7% 11/576 vs 10/579
Major GU bleeding (DOAC vs LMWH)	1.0% vs 0% 5/522 vs 0/524	0.5% vs 0% 1/203 vs 0/203	0% vs 0% 0/145 vs 0/142	0.7% vs 0.2% 4/572 vs 1/578
Intracranial bleeding	0.4% vs 0.8% 2/522 vs 4/524	0% vs 0% 0/203 vs 0/203	0% vs 0.7% 0/145 vs 1/142	0% vs 0.3% 0/576 vs 2/579
Fatal Bleeding (DOAC vs LMWH)	0% vs 0.4% 0/522 vs 2/524	0.5% vs 0.5% 1/203 vs 1/203	0% vs 0% 0/145 vs 0/142	0% vs 0.3% 0/576 vs 2/579
Fatal PE^b^ (DOAC vs LMWH)	1.1% vs 0.7% 6/522 vs 4/524	0.5% vs 0.5% 1/203 vs 1/203	0% vs 0% 0/203 vs 0/203	0.7% vs 0.5% 4/576 vs 3/579
Treatment discontinuation^c^ (DOAC vs LMWH)	12 mo: 61.7% vs 70.6% 322 vs 370 6 mo: 41.2% vs 45.6% 219 vs 239	42.4% vs 44.3% 86 vs 90	37.9% vs 45.8% 55 vs 65	36.8% vs 44.6% 212 vs 258

Note

Outcomes rates are obtained at 12 mo of follow‐up for Hokusai VTE Cancer and at 6 mo of follow up for SELECT‐D, ADAM VTE, and Caravaggio.

CRNMB, clinically relevant nonmajor bleeding; DOAC, direct oral anticoagulation; GI, gastrointestinal; GU, genitourinary; HR, hazard ratio; IU/kg, international units per kilogram of bodyweight; LMWH, low‐molecular‐weight heparin; mITT, modified intention to treat population; PE, pulmonary embolism; VTE, venous thromboembolism.

^a^ Modified intention to treat for Hokusai VTE Cancer, SELECT D, and Caravaggio. Per protocol for ADAM VTE.

^b^ Including death for which PE could not be ruled out as a cause, as defined within respective studies.

^c^ Treatment discontinuation comprises all patients who did not receive either drug for the predefined time (including deceased patients).

Overall, 2894 patients with acute VTE were included in the meta‐analysis. Of those, 1446 were allocated to treatment with a DOAC and 1448 to LMWH (50% each). Incidental VTE was reported as the index event in 30.0% of patients, ranging from 19.9% in Caravaggio, 27.8% in SELECT‐D to 32.5% in Hokusai VTE Cancer, with no reported rates of presence or absence of symptoms of the qualifying VTE events in ADAM VTE.^12^, ^13^, ^14^, ^15^

Patients allocated to the control group of all 4 studies were treated with dalteparin at a dose of 200 IU/kg once daily for 1 month, followed by 150 IU/kg once daily. Experimental treatment was edoxaban at a dose of 60 mg once daily (or 30 mg if [i] body weight was below 60 kg, [ii] creatinine clearance between 30 and 50 mg/min, or [iii] in the case of concomitant treatment with a potent P‐glycoprotein inhibitor) after 5 days of therapeutic anticoagulation with LMWH in the Hokusai VTE Cancer study, rivaroxaban 15 mg twice daily for 3 weeks followed by 20 mg once daily in SELECT‐D, and apixaban 10 mg twice daily for 7 days followed by 5 mg twice daily in the ADAM VTE and Caravaggio trials. The duration of treatment differed among studies; however, all studies reported 6‐month rates of outcome variables. The primary outcome of Hokusai VTE Cancer was a composite of recurrent VTE and major bleeding at 12 months, recurrent VTE at 6 months for SELECT‐D and Caravaggio, and major bleeding at 6 months for ADAM VTE.^12^, ^13^, ^14^, ^15^ Six‐month rates of outcome events were derived from the main manuscripts of included trials (SELECT‐D, ADAM VTE, Caravaggio) or supplemental files (Hokusai VTE Cancer).

### 3.3 Risk of bias

**FIGURE 2:**
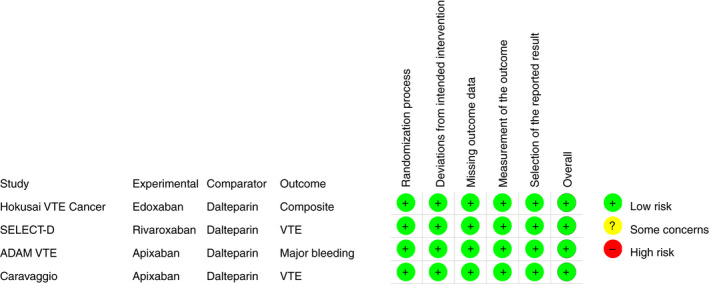
Revised Cochrane risk‐of‐bias tool (RoB2) for randomized trials. VTE, venous thromboembolism

Risk of bias was assessed by the Revised Cochrane risk‐of‐bias tool for randomized trials.^21^ Low risk of bias was suspected for all included studies (Figure 2). Visual inspection of a funnel plot revealed no suspicion for publication bias (Figure S1).

### 3.4 Primary outcomes of the meta‐analysis: recurrent VTE and major bleeding

**FIGURE 3:**
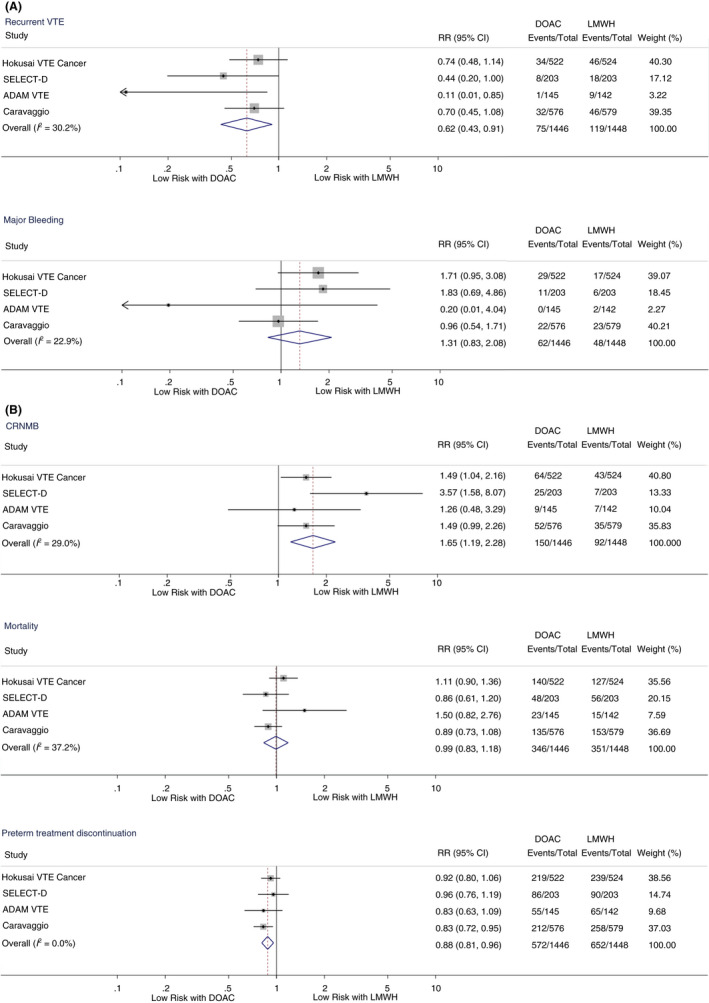
(A) Forest plots and relative risks for primary efficacy and safety outcomes. CI, confidence interval; DOAC, direct oral anticoagulant, LMWH, low‐molecular‐weight heparin; RR, relative risk; VTE, venous thromboembolism. (B) Forest plots and relative risks for key secondary outcomes. CI, confidence interval; CRNMB, clinically relevant nonmajor bleeding; DOAC, direct oral anticoagulant; LMWH, low‐molecular‐weight heparin; RR, relative risk

The 6‐month risk of recurrent VTE was significantly reduced in patients treated with a DOAC compared to LMWH (RR, 0.62 [95% CI, 0.43‐0.91]; *P* = .014; I^2^ = 30.2%). Recurrent VTE during 6 months of follow‐up occurred in 75 of 1446 (5.2%) patients treated with DOACs and 119 of 1448 (8.2%) patients treated with LMWHs. Conversely, risk of major bleeding was higher in patients treated with DOACs (RR 1.31 [95% CI, 0.83‐1.71]; *P* = .252; I^2^ = 22.9%), with rates of major bleeding of 4.3% and 3.3% in patients treated with DOACs and LMWHs, respectively. Forest plots and corresponding RRs of the primary outcome analysis are displayed in Figure 3A.

### 3.5 Secondary outcomes of the meta‐analysis

Risk of CRNMB was significantly elevated in patients treated with DOACs compared to LMWHs (RR, 1.65 [95% CI, 1.19‐2.28]; *P* = .002; I^2^ = 29.0%), with corresponding 6‐month rates of 10.4% and 6.4%, respectively. Accordingly, rates of any bleeding event (major bleeding and/or CRNMB) were increased in patients treated with DOACs versus LMWHs (14.7% vs 9.7%; RR 1.54 [95% CI, 1.12‐2.11]; *P* = .007; I^2^ = 48.3%). There was a nonsignificant increase in risk of major GI bleeding in patients treated with DOACs (2.6% vs 1.4%; RR, 1.85 [95% CI, 0.92‐3.71]; *P* = .083; I^2^ = 35.3%) and an increase in risk of major GU bleeding (0.7% vs 0.1%; RR, 4.99 [95% CI, 1.08‐23.08]; *P* = .040; I^2^ = 0.0%).

There was no difference in mortality among study groups (RR, 0.99 [95% CI, 0.83‐1.18]; *P* = .928; I^2^ = 37.2%), with corresponding 6‐month mortality rates of 23.9% in patients treated with DOACs and 24.2% in those treated with LMWHs. Also, no significant differences between DOACs and LMWHs were seen with regards to PE‐related mortality (0.7% vs 0.5%; RR, 1.43 [95% CI, 0.55‐3.76]; *P* = .467; I^2^ = 0.0%), fatal bleeding events (0.1% vs 0.3%; RR, 0.37 [95% CI, 0.07‐2.00]; *P* = .247; I^2^ = 0.0%), or intracranial bleeding (0.1% vs 0.5%; RR, 0.39 [95% CI, 0.10‐1.49]; *P* = .168; I^2^ = 0.0%).

The rate of preterm discontinuation of anticoagulation was significantly lower in patients treated with DOACs compared to LMWHs (39.6% vs 45.0%; RR, 0.88 [95% CI, 0.81‐0.96]; *P* = .003; I^2^ = 0.0%).

For the analysis of major GI bleeding, major GU bleeding, fatal bleeding events, and intracranial bleeding, 12‐month rates from Hokusai VTE Cancer were used due to the unavailability of 6‐month rates.

Figure 3B depicts forest plots and corresponding RR for key secondary outcome events (CRNMB, mortality, treatment discontinuation). Forest plots for the remaining secondary outcomes (any bleeding, fatal bleeding, fatal PE, major GI bleeding, major GU bleeding, and intracranial bleeding are provided in the Appendix S1 (Figure S2).

### 3.6 Sensitivity analyses

Upon visual inspection of forest plots and I^2^ values, heterogeneity in outcomes was deemed possible between ADAM VTE and the other included studies. This heterogeneity is further supported by an overall lower event rate of VTE and bleeding as well as lower overall mortality rates of patients in ADAM VTE compared to the Hokusai VTE Cancer, SELECT‐D, and Caravaggio studies, indicating the recruitment of a presumably low‐risk population. To reduce heterogeneity, we conducted a sensitivity analysis by aggregating outcomes of Hokusai VTE Cancer, SELECT‐D, and Caravaggio. Similar results were obtained for risk of outcome events for patients treated with DOACs compared to LMWHs, while heterogeneity was reduced for most outcomes (recurrent VTE: RR, 0.68 [95% CI, 0.51‐0.90]; I^2^ = 0.0%; major bleeding: RR, 1.36 [95% CI, 0.89‐2.06]; I^2^ = 15.2%; CRNMB: RR, 1.74 [95% CI, 1.17‐2.59]; I^2^ = 49.6%; mortality: RR, 0.96 [95% CI, 0.82‐1.13]; I^2^ = 29.6%). Forest plots of the sensitivity analysis are provided as Figure S3.

Within a second sensitivity analysis, we explored risk of recurrent VTE and major bleeding of patients treated with DOAC compared to LMWH during the on‐treatment period. Only data for Caravaggio and the 12‐month period of Hokusai VTE Cancer were available and were aggregated within a pooled analysis due to the unavailability of corresponding data for SELECT‐D and ADAM VTE. RR for recurrent VTE (4.8% vs 7.6%; RR, 0.63 [95% CI, 0.45‐0.89]; *P* = .009; I^2^ = 0.0%) and major bleeding (4.8% vs 3.6%; RR, 1.32 [95% CI, 0.66‐2.63]; *P* = .426; I^2^ = 35.9%) were comparable to the primary analysis (Figure S4).

### 3.7 Subgroup analyses

We conducted a subgroup analysis of risk of major bleeding in patients with GI cancer, including colorectal, gastric, gastroesophageal, pancreatic, and hepatobiliary cancer. Detailed data for a 6‐month observation period were available for Hokusai VTE Cancer and SELECT‐D (aggregated n = 1452; 33% GI cancer).^12^, ^13^, ^22^

Risk of major bleeding was significantly elevated in patients with GI cancer treated with DOACs compared to LMWHs (9.3% vs 4.0%; RR, 2.30 [95% CI, 1.08‐4.88]; *P* = .031; I^2^ = 0.0%). Conversely, in patients with non‐GI malignancies, risk of major bleeding in patients treated with DOACs vs LMWHs was comparable (3.4% vs 2.9%; RR, 1.22 [95% CI, 0.60‐2.48]; *P* = .580; I^2^ = 0.0%).

**FIGURE 4:**
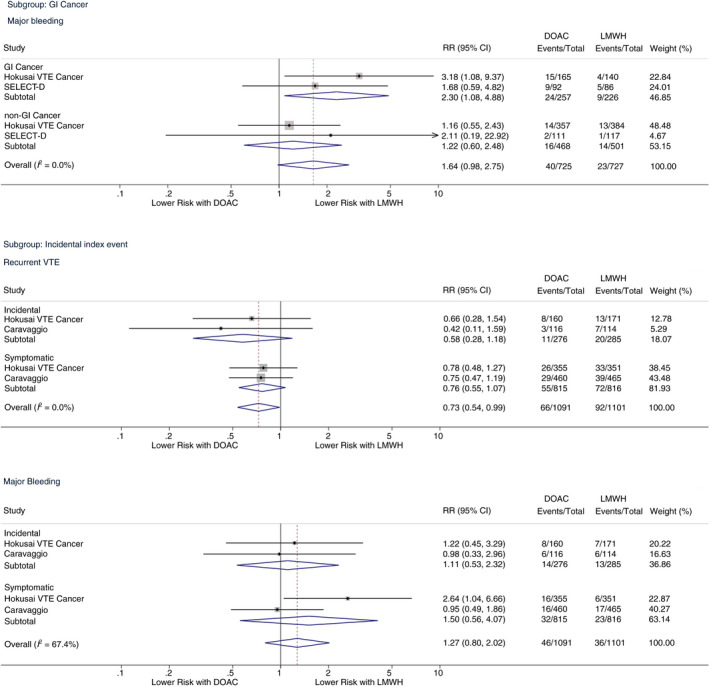
Subgroup analyses. CI, confidence interval; DOAC, direct oral anticoagulant; GI, gastrointestinal; LMWH, low molecular weight heparin; RR, risk ratio; VTE, venous thromboembolism

We also analyzed efficacy and safety outcomes in the subgroup of patients with incidental VTE as the index event. Data for outcome variables stratified by symptoms of the index event were available for Hokusai VTE Cancer and Caravaggio (aggregated n = 2192; 26% incidental VTE).^12^, ^14^, ^23^ Risk of recurrent VTE was similarly reduced in patients treated with DOACs with incidental and symptomatic VTE as the index event (incidental: RR, 0.58 [95% CI, 0.28‐1.18]; *P* = .134; I^2^ = 0.0%; symptomatic: RR, 0.76 [95% CI, 0.55‐1.07]; *P* = .118; I^2^ = 0.0%). Risk of major bleeding was similar between patients with incidental and symptomatic VTE as the index event (incidental: RR, 1.11 [95% CI, 0.53‐2.32]; *P* = .785; I^2^ = 0.0%; symptomatic: RR 1.50 [95% CI, 0.55‐4.07]; *P* = .422; I^2^ = 67.4%). Figure 4 displays detailed results of both subgroup analyses.

## 4 DISCUSSION

In this meta‐analysis of 4 RCTs including 2894 patients, we compared the efficacy and safety of DOACs vs LMWHs for the treatment of cancer‐associated VTE. DOACs emerged as significantly more effective in preventing recurrent VTE than LMWHs, while RRs of bleeding were in favor of LMWHs.

Superiority with respect to recurrent VTE has not been shown for a DOAC, neither in any of the single RCTs nor in aggregated results of previous meta‐analyses.^16^, ^17^ The improvement in efficacy of DOACs over LMWHs might be explained in part by better treatment adherence with an orally administered drug than with subcutaneous injections, which may be reflected by a lower preterm treatment discontinuation rate in patients treated with oral factor Xa inhibitors. Further advantages could be the predictable effect of DOACs and no need for laboratory drug monitoring or frequent dose modifications of oral treatment strategies. The impact of the lower treatment discontinuation rate with DOACs was explored in a pooled analysis of outcomes for the on‐treatment period of Hokusai VTE Cancer and Caravaggio. Efficacy and safety of this analysis were comparable to the primary analysis, arguing against treatment adherence being the only driving factor for reduced rates of recurrent VTE with DOACs.

Another reason for the relative improvement in efficacy of DOACs versus LMWHs could be the reduction of the dosing of LMWHs in the control arm by 25% after 1 month of treatment, as represented by diverging Kaplan‐Meier curves from 1‐2 months of treatment initiation in the trials.^12^, ^13^, ^14^, ^15^ The risk reduction of recurrent VTE with DOACs seems to be independent of differences of qualifying events for study inclusion with regard to location of the thrombosis, with splanchnic and cerebral vein thrombosis being included in the ADAM VTE trial, and definition of efficacy outcome events, with upper extremity DVT being included in the Caravaggio trial.^14^, ^15^ Based on the low level of heterogeneity and consistency of efficacy results of DOACs between studies included in the meta‐analysis, as represented by similar patterns of forest plots upon visual inspection and low I^2^ values (ie, the percentage of variation in RR attributable to differences between studies), it appears reasonable to come to the general conclusion that factor Xa inhibitors are effective in preventing recurrent VTE.

On the contrary, DOACs seem to be associated with an increased risk of bleeding. We found a numerically elevated rate of major bleeding and major GI bleeding in patients treated with DOACs, with a significantly elevated risk of major bleeding in the subgroup of patients with a GI cancer. Further, risk of major GU bleeding and risk of CRNMB was significantly higher in patients treated with a DOAC. However, rates of intracranial and fatal bleeding were low across all studies in both treatment arms, with comparable rates of intracranial and fatal bleeding between DOAC and LMWH arms (0.1% vs 0.5% and 0.1% vs 0.3%, respectively), which supports the relative safety profile of DOACs. Despite differences between the rates of bleeding events across different factor Xa inhibitors, similar results could be observed between the respective dalteparin control arms, raising the question of whether differences in potential safety profiles are agent specific or trial specific.

Similar results for safety and efficacy were obtained in 2 subgroup analyses. In patients with GI malignancies, risk of major bleeding was significantly higher in the DOAC than in the LMWH treatment arm. Specifics on type of major bleeding in patients with GI cancer were not available for SELECT‐D, ADAM VTE, and Caravaggio. However, Kraaijpoel et al^26^ reported a high proportion of major GI bleeding of all major bleeding events in the subgroup of patients with GI cancer treated with DOACs (90%) compared to LMWHs (20%) for Hokusai VTE Cancer. In our second subgroup analysis of patients with incidental VTE as the index event, risk of recurrent VTE and major bleeding of DOACs compared to LMWHs were comparable to outcomes in patients with symptomatic VTE as the index event. This observation supports current recommendations for the same therapeutic approach for incidental and symptomatic cancer‐associated VTE.^28^, ^29^

Despite a higher efficacy and better therapy adherence, DOACs were not associated with a decreased mortality rate compared to LMWHs. Rates of fatal PE and fatal bleeding were both low between groups, and therefore these analyses might lack statistical power. It is also likely that underlying cancer‐related mortality overwhelms potential survival benefits of DOACs by reducing risk of recurrent VTE.

Due to heterogeneity in overall outcome rates and differences in qualifying VTE events, design and outcome events of the ADAM VTE trial, we conducted a sensitivity analysis including only data from Hokusai VTE Cancer, SELECT‐D, and Caravaggio, where comparable results to the primary analyses were found.

Our updated meta‐analysis has several limitations. First, data synthesis was conducted from published results only and does not include patient‐level data. Therefore, additional subgroup analyses were not possible that would be feasible within a patient‐level meta‐analysis. However, all primary and secondary outcome analyses were feasible in our study‐level meta‐analysis. Second, despite dalteparin being an evidence‐based treatment for cancer‐associated VTE based on the CLOT study used as comparator in all 4 trials, other LMWHs with different dosing strategies are also used in clinical practice in many countries. Third, due to the unfeasibility of placebo injections over at least 6 months, all 4 studies included in the meta‐analysis were conducted with an open‐label design. Certainly, this represents the ethically preferable study design, despite a certain degree of potential risk of bias due to lack of blinding. This issue is largely resolved by blinded‐endpoint evaluation, as conducted within the studies. Fourth, we performed 2 subgroup analyses of special interest, and in the subgroup analysis of GI cancers, only 2 studies with extractable information could be included; in the subgroup analysis of patients stratified by the index event (incidental or symptomatic), data from only the 2 largest studies were available. Thus, our subgroup analyses are limited in sample size and therefore might lack statistical power. Finally, for the analysis of our secondary outcomes, major GI bleeding, fatal bleeding events, and intracranial bleeding, 12‐month rates from Hokusai VTE Cancer were used as of the unavailability of 6‐month rates. However, as of the low overall event rates of these specific outcomes and their clinical significance, we decided to aggregate these data despite heterogeneity in their analytic time frame. For all other primary and secondary outcome analysis, 6‐month rates from all studies were used.

One strength of our meta‐analysis is its scale, with the recent publication of the Caravaggio trial, adding a significant number of patients for aggregating data. Thereby, the power is higher compared to previous meta‐analyses, which enhances the generalizability of the results. Further, updated guideline recommendations for the treatment of cancer‐associated VTE, naming DOACs as alternative to LMWHs as first‐line therapeutic option, are supported by our meta‐analysis.^29^, ^30^, ^31^ Due to the improved efficacy reported in our meta‐analysis and its practical advantages beyond efficacy and safety leading to improved patient adherence, superiority of DOACs to LMWHs in the setting of acute cancer‐associated VTE should be evaluated in updated guidelines in the future.

## 5 CONCLUSION

In patients with cancer‐associated VTE, DOACs significantly reduce risk of recurrent VTE and increase numerically the risk of major bleeding in a meta‐analysis of 4 RCTs comparing factor Xa inhibitors to dalteparin. DOACs were further associated with an increased risk of CRNMB in the overall patient population and an increased risk of major bleeding in the subgroup of patients with GI cancer, respectively. Therefore, the decision for a specific anticoagulant for the treatment of VTE in patients with cancer should be personalized. Optimal decision making should include a careful balancing of risks and benefits of the available treatment options and patient preferences.

## RELATIONSHIP DISCLOSURE

FM reports nothing to disclose. FP has received honoraria for lectures and advisory board meetings from Daiichi‐Sankyo, BMS/Pfizer, and MSD Oncology. CZ has received consultancies and speaker’s honoraria from Roche, Novartis, BMS, MSD, Imugene, Ariad, Pfizer, Merrimack, Merck KGaA, Fibrogen, AstraZeneca, Tesaro, Gilead, Servier, Shire, Eli Lilly, and Athenex. IP has received occasional honoraria for lectures and advisory board meetings from Bayer AG, Daiichi Sankyo, Boehringer Ingelheim, Sanofi, and BMS/Pfizer. CA has received honoraria for lectures from Bayer, Daiichi‐Sankyo, BMS/Pfizer, and Sanofi; and articipation in advisory boards for Bayer, Boehringer‐Ingelheim, Daiichi‐Sankyo, and BMS/Pfizer.

## AUTHOR CONTRIBUTIONS

FM: conceptualization, data curation, formal analysis, methodology, and writing of the original draft. FP: conceptualization, formal analysis, methodology, –review, and editing. CZ: supervision, –review, and editing. IP: project administration, supervision, –review, snd editing. CA: conceptualization, project administration, supervision, and writing the original draft.

## Supporting information

Appendix S1

Appendix S2

## References

[1] Horsted F , West J , Grainge MJ . Risk of venous thromboembolism in patients with cancer: a systematic review and meta‐analysis. PLoS Med. 2012;9(7):e1001275. 22859911 10.1371/journal.pmed.1001275 PMC3409130

[2] Khorana AA , Francis CW , Culakova E , Kuderer NM , Lyman GH . Thromboembolism is a leading cause of death in cancer patients receiving outpatient chemotherapy. J Thrombosis Haemostasis: JTH. 2007;5(3):632–4. 10.1111/j.1538-7836.2007.02374.x 17319909

[3] Ay C , Pabinger I , Cohen AT . Cancer‐associated venous thromboembolism: burden, mechanisms, and management. Thromb Haemost. 2017;117(2):219–30. 27882374 10.1160/TH16-08-0615

[4] Cohen AT , Katholing A , Rietbrock S , Bamber L , Martinez C . Epidemiology of first and recurrent venous thromboembolism in patients with active cancer. A population‐based cohort study. Thrombosis Haemostasis. 2017;117(1):57–65. 27709226 10.1160/TH15-08-0686

[5] Prandoni P , Lensing AWA , Piccioli A , Bernardi E , Simioni P , Girolami B , et al. Recurrent venous thromboembolism and bleeding complications during anticoagulant treatment in patients with cancer and venous thrombosis. Blood. 2002;100(10):3484. 12393647 10.1182/blood-2002-01-0108

[6] Lee AYY , Levine MN , Baker RI , Bowden C , Kakkar AK , Prins M , et al. Low‐molecular‐weight heparin versus a coumarin for the prevention of recurrent venous thromboembolism in patients with cancer. N Engl J Med. 2003;349(2):146–53. 12853587 10.1056/NEJMoa025313

[7] Ay C , Kamphuisen PW , Agnelli G . Antithrombotic therapy for prophylaxis and treatment of venous thromboembolism in patients with cancer: review of the literature on current practice and emerging options. ESMO Open. 2017;2(2):e000188. 28761749 10.1136/esmoopen-2017-000188 PMC5519804

[8] Posch F , Konigsbrugge O , Zielinski C , Pabinger I , Ay C . Treatment of venous thromboembolism in patients with cancer: a network meta‐analysis comparing efficacy and safety of anticoagulants. Thromb Res. 2015;136(3):582–9. 26210891 10.1016/j.thromres.2015.07.011 PMC7311195

[9] Kearon C , Akl EA , Ornelas J , Blaivas A , Jimenez D , Bounameaux H , et al. Antithrombotic therapy for VTE disease: CHEST guideline and expert panel report. Chest. 2016;149(2):315–52. 26867832 10.1016/j.chest.2015.11.026

[10] Konstantinides SV , Meyer G , Becattini C , Bueno H , Geersing G‐J , Harjola V‐P , et al. 2019 ESC Guidelines for the diagnosis and management of acute pulmonary embolism developed in collaboration with the European Respiratory Society (ERS): The Task Force for the diagnosis and management of acute pulmonary embolism of the European Society of Cardiology (ESC). Eur Heart J. 2019;41(4):543–603. 10.1093/eurheartj/ehz405 31504429

[11] Witt DM , Nieuwlaat R , Clark NP , Ansell J , Holbrook A , Skov J , et al. American Society of Hematology 2018 guidelines for management of venous thromboembolism: optimal management of anticoagulation therapy. Blood Adv. 2018;2(22):3257–91. 30482765 10.1182/bloodadvances.2018024893 PMC6258922

[12] Raskob GE , van Es N , Verhamme P , Carrier M , Di Nisio M , Garcia D , et al. Edoxaban for the treatment of cancer‐associated venous thromboembolism. N Engl J Med. 2018;378(7):615–24. 29231094 10.1056/NEJMoa1711948

[13] Young AM , Marshall A , Thirlwall J , Chapman O , Lokare A , Hill C , et al. Comparison of an oral factor Xa inhibitor with low molecular weight heparin in patients with cancer with venous thromboembolism: results of a randomized trial (SELECT‐D). J Clin Oncol. 2018;36(20):2017–23. 29746227 10.1200/JCO.2018.78.8034

[14] Agnelli G , Becattini C , Meyer G , Muñoz A , Huisman MV , Connors JM , et al. Apixaban for the treatment of venous thromboembolism associated with cancer. N Engl J Med. 2020;382(17):1599–1607. 32223112 10.1056/NEJMoa1915103

[15] McBane R II , Wysokinski WE , Le‐Rademacher JG , Zemla T , Ashrani A , Tafur A , et al. Apixaban and dalteparin in active malignancy associated venous thromboembolism: the ADAM VTE trial. J Thromb Haemost. 2020;18(2):411–21. 31630479 10.1111/jth.14662

[16] Mai V , Tanguay VF , Guay CA , Bertoletti L , Magnan S , Turgeon AF , et al. DOAC compared to LMWH in the treatment of cancer related‐venous thromboembolism: a systematic review and meta‐analysis. J Thromb Thrombolysis. 2020 10.1007/s11239-020-02055-1 32052314

[17] Li A , Garcia DA , Lyman GH , Carrier M . Direct oral anticoagulant (DOAC) versus low‐molecular‐weight heparin (LMWH) for treatment of cancer associated thrombosis (CAT): a systematic review and meta‐analysis. Thromb Res. 2019;173:158–63. 29506866 10.1016/j.thromres.2018.02.144 PMC6119655

[18] Rossel A , Robert‐Ebadi H , Combescure C , Grosgurin O , Stirnemann J , Addeo A , et al. Anticoagulant therapy for acute venous thrombo‐embolism in cancer patients: a systematic review and network meta‐analysis. PLoS ONE. 2019;14(3):e0213940. 30897142 10.1371/journal.pone.0213940 PMC6428324

[19] Vedovati MC , Giustozzi M , Bonitta G , Agnelli G , Becattini C . Efficacy and safety of anticoagulant agents in patients with venous thromboembolism and cancer: a network meta‐analysis. Thromb Res. 2018;170:175–80. 30196195 10.1016/j.thromres.2018.08.023

[20] Higgins JPTTJ , Chandler J , Cumpston M , Li T , Page MJ , Welch VA , editors. Cochrane handbook for systematic reviews of interventions version 6.0 (updated July 2019). Cochrane; 2019.

[21] Higgins JP , Altman DG , Gotzsche PC , Juni P , Moher D , Oxman AD , et al. The Cochrane Collaboration's tool for assessing risk of bias in randomised trials. BMJ. 2011;343:d5928. 22008217 10.1136/bmj.d5928 PMC3196245

[22] Mulder FI , van Es N , Kraaijpoel N , Di Nisio M , Carrier M , Duggal A , et al. Edoxaban for treatment of venous thromboembolism in patient groups with different types of cancer: results from the Hokusai VTE Cancer study. Thromb Res. 2020;185:13–9. 31733403 10.1016/j.thromres.2019.11.007

[23] Mulder FI , Di Nisio M , Ay C , Carrier M , Bosch FTM , Segers A , et al. Clinical implications of incidental venous thromboembolism in cancer patients. Eur Respir J. 2020;55(2):1901697. 31727694 10.1183/13993003.01697-2019

[24] Di Nisio M , van Es N , Carrier M , Wang TF , Garcia D , Segers A , et al. Extended treatment with edoxaban in cancer patients with venous thromboembolism: a post‐hoc analysis of the Hokusai‐VTE Cancer study. J Thrombosis Haemostasis: JTH. 2019;17(11):1866–74. 10.1111/jth.14561 31271705

[25] Marshall A , Levine M , Hill C , Hale D , Thirlwall J , Wilkie V , et al. Treatment of cancer‐associated venous thromboembolism: 12‐month outcomes of the placebo versus rivaroxaban randomization of the SELECT‐D Trial (SELECT‐D: 12m). J Thrombosis Haemostasis: JTH. 2020;18(4):905–15. 10.1111/jth.14752 31995662

[26] Kraaijpoel N , Di Nisio M , Mulder FI , van Es N , Beyer‐Westendorf J , Carrier M , et al. Clinical impact of bleeding in cancer‐associated venous thromboembolism: results from the Hokusai VTE cancer study. Thromb Haemost. 2018;118(8):1439–49. 30060256 10.1055/s-0038-1667001

[27] Moher D , Liberati A , Tetzlaff J , Altman DG; the PRISMAGroup . Preferred reporting items for systematic reviews and meta‐analyses: the PRISMA statement. Ann Intern Med. 2009;151(4):264–9. 19622511 10.7326/0003-4819-151-4-200908180-00135

[28] Di Nisio M , Lee AYY , Carrier M , Liebman HA , Khorana AA , et al ; the Subcommittee on Haemostasis and Malignancy . Diagnosis and treatment of incidental venous thromboembolism in cancer patients: guidance from the SSC of the ISTH. J Thromb Haemost. 2015;13(5):880–3. 25714858 10.1111/jth.12883

[29] Key NS , Khorana AA , Kuderer NM , Bohlke K , Lee AYY , Arcelus JI , et al. Venous thromboembolism prophylaxis and treatment in patients with cancer: ASCO clinical practice guideline update. J Clin Oncol. 2019;JCO.19.01461.38(5):496–520. 31381464 10.1200/JCO.19.01461

[30] Khorana AA , Noble S , Lee AYY , Soff G , Meyer G , O'Connell C , et al. Role of direct oral anticoagulants in the treatment of cancer‐associated venous thromboembolism: guidance from the SSC of the ISTH. J Thrombosis Haemostasis: JTH. 2018;16(9):1891–4. 10.1111/jth.14219 30027649

[31] Farge D , Frere C , Connors JM , Ay C , Khorana AA , Munoz A , et al. 2019 international clinical practice guidelines for the treatment and prophylaxis of venous thromboembolism in patients with cancer. Lancet Oncol. 2019;20(10):e566 –81. 31492632 10.1016/S1470-2045(19)30336-5

