# Supplementary material for: Direct oral anticoagulants compared to low‐molecular‐weight heparin for the treatment of cancer‐associated thrombosis: Updated systematic review and meta‐analysis of randomized controlled trials
Source: Res Pract Thromb Haemost. 2020 May 21;4(4):550–61. doi: 10.1002/rth2.12359 (PMC7292654; doi:10.1002/rth2.12359)

**Direct oral anticoagulants compared to low-molecular-weight-heparin for treatment of cancer-associated thrombosis: Updated systematic review and meta-analysis of randomized controlled trials**

**Supplemental File**

# Search strategy

## EMBASE

#1 'neoplasms'/exp

#2 'cancer*':ti,ab OR 'carcinoma*’:ti,ab OR 'neoplas*’:ti,ab OR 'tumour*’:ti,ab OR 'tumor*’:ti,ab OR 'malign*’:ti,ab

#3 'venous thromboembolism’/exp

#4 'venous thrombosis’/exp

#5 'pulmonary embolism’/exp

#6 'thrombo*’:ti,ab OR 'venous thromb*’:ti,ab OR 'VTE*’:ti,ab

#7 'Factor Xa Inhibitors’/exp

#8 'apixaban*’:ti,ab OR 'edoxaban*’:ti,ab OR 'rivaroxaban*’:ti,ab OR 'dabigatran*’:ti,ab OR 'DOAC*’:ti,ab OR 'direct oral anticoagulant*’:ti,ab OR 'NOAC*’:ti,ab OR 'new oral anticoagulant*’:ti,ab OR 'non-vitamin K oral anticoagulant*’:ti,ab

#9 'Heparin, Low-Molecular-Weight’/exp

#10 'dalteparin’/exp

#11 'enoxaparin’/exp

#12 'nadroparin’/exp

#13 'tinzaparin’/exp

#14 'LMWH*’:ti,ab OR 'low molecular weight heparin*’:ti,ab OR 'enoxaparin*’:ti,ab OR 'dalteparin*’:ti,ab OR 'nadroparin*’:ti,ab OR 'tinzaparin*’:ti,ab

#15 (#1 OR #2) AND (#3 OR #4 OR #5 OR #6) AND (#7 OR #8) AND (#9 OR #10 OR #11 OR #12 OR #13 OR #14)

**Utilized filters:**

- 'clinical study'/de OR 'comparative effectiveness'/de OR 'comparative study'/de OR 'controlled clinical trial'/de OR 'controlled study'/de OR 'double blind procedure'/de OR 'major clinical study'/de OR 'multicenter study'/de OR 'multicenter study topic'/de OR 'phase 2 clinical trial topic'/de OR 'phase 3 clinical trial topic'/de OR 'randomized controlled trial'/de OR 'randomized controlled trial topic'/de) AND 'human'/de
- 'article'/it OR 'article in press'/it OR 'letter'/it

**396 results**

## MEDLINE (via PubMed):

((neoplasms[mesh]) OR (cancer*[tiab] OR carcinoma*[tiab] OR neoplas*[tiab] OR tumour*[tiab] OR tumor*[tiab] OR malign*[tiab]))

AND

((venous thromboembolism[mesh] OR venous thrombosis[mesh] OR pulmonary embolism[mesh]) OR (thrombo*[tiab] OR venous thromb*[tiab] OR VTE*[tiab]))

AND

((Factor Xa Inhibitors[mesh]) OR (apixaban*[tiab] OR edoxaban*[tiab] OR rivaroxaban*[tiab] OR dabigatran*[tiab] OR DOAC*[tiab] OR direct oral anticoagulant*[tiab] OR NOAC*[tiab] OR new oral anticoagulant*[tiab] OR non-vitamin K oral anticoagulant*[tiab]))

AND

((Heparin, Low-Molecular-Weight[mesh] OR dalteparin[mesh] OR enoxaparin[mesh] OR nadroparin[mesh] OR tinzaparin[mesh]) OR (LMWH*[tiab] OR low molecular weight heparin*[tiab] OR enoxaparin*[tiab] OR dalteparin*[tiab] OR nadroparin*[tiab] OR tinzaparin*[tiab]))

**Filters**: Humans, Clinical trial

**216 results**

## CENTRAL

#1 MeSH descriptor: [Neoplasms] explode all trees

#2 cancer* OR carcinoma* OR neoplas* OR tumour* OR tumor* OR malign*

#3 MeSH descriptor: [Venous Thrombosis] explode all trees

#4 MeSH descriptor: [Venous Thromboembolism] explode all trees

#5 MeSH descriptor: [Pulmonary Embolism] explode all trees

#6 thrombo* OR venous thromb* OR VTE*

#7 MeSH descriptor: [Factor Xa Inhibitors] explode all trees

#8 apixaban* OR edoxaban* OR rivaroxaban* OR dabigatran* OR DOAC* OR direct oral anticoagulant* OR NOAC* OR new oral anticoagulant* OR non-vitamin K oral anticoagulant*

#9 MeSH descriptor: [Heparin, Low-Molecular-Weight] explode all trees

#10 MeSH descriptor: [Dalteparin] explode all trees

#11 MeSH descriptor: [Enoxaparin] explode all trees

#12 MeSH descriptor: [Nadroparin] explode all trees

#13 MeSH descriptor: [Tinzaparin] explode all trees

#14 LMWH* OR low molecular weight heparin* OR enoxaparin* OR dalteparin* OR nadroparin* OR tinzaparin*

(#1 OR #2) AND (#3 OR #4 OR #5 OR #6) AND (#7 OR #8) AND (#9 OR #10 OR #11 OR #12 OR #13 OR #14)

**Filter**: Trials

**147 results**

## Supplemental Figure S1: Funnel plot for primary efficacy outcome (VTE) of included randomized controlled trials (asymmetry indicates publication bias)


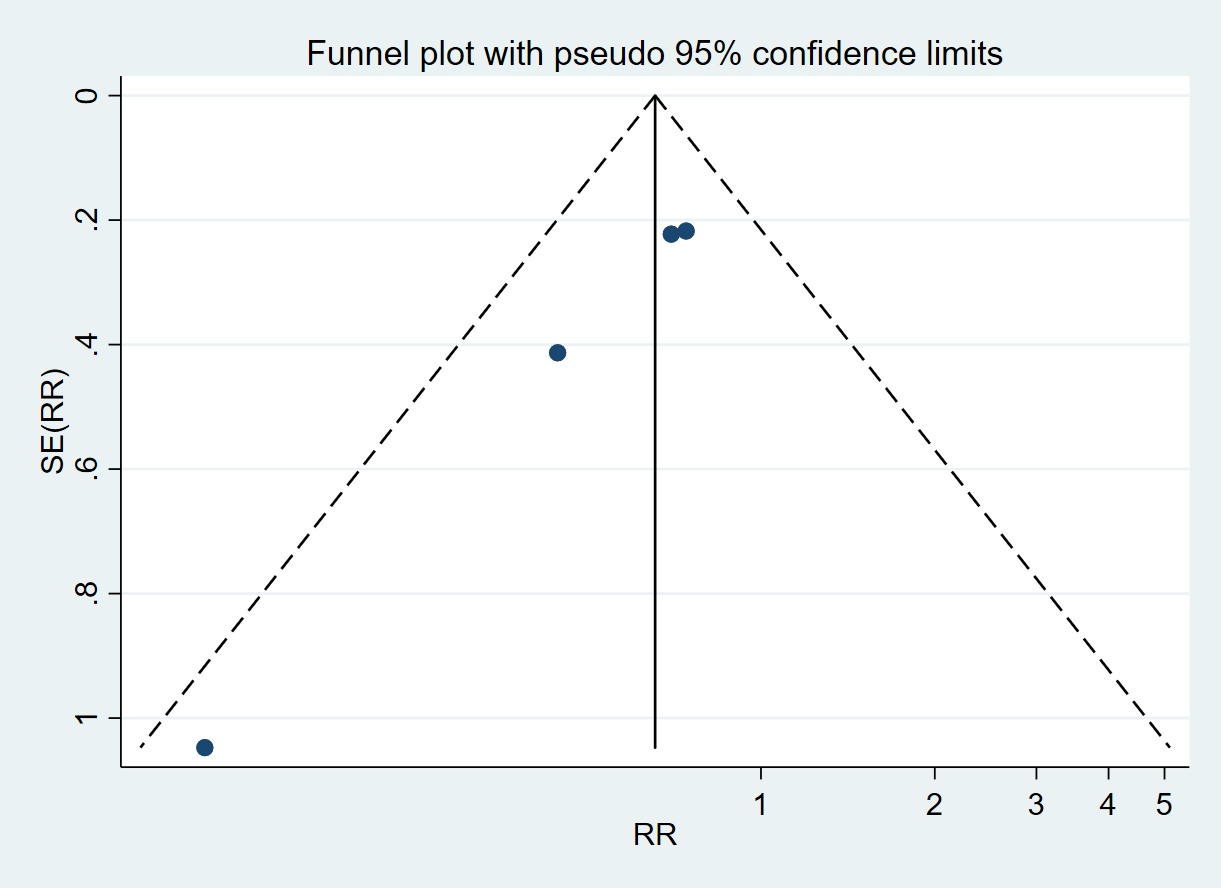


## Supplemental Figure S2: Forrest plots and risk ratios of secondary outcome variables
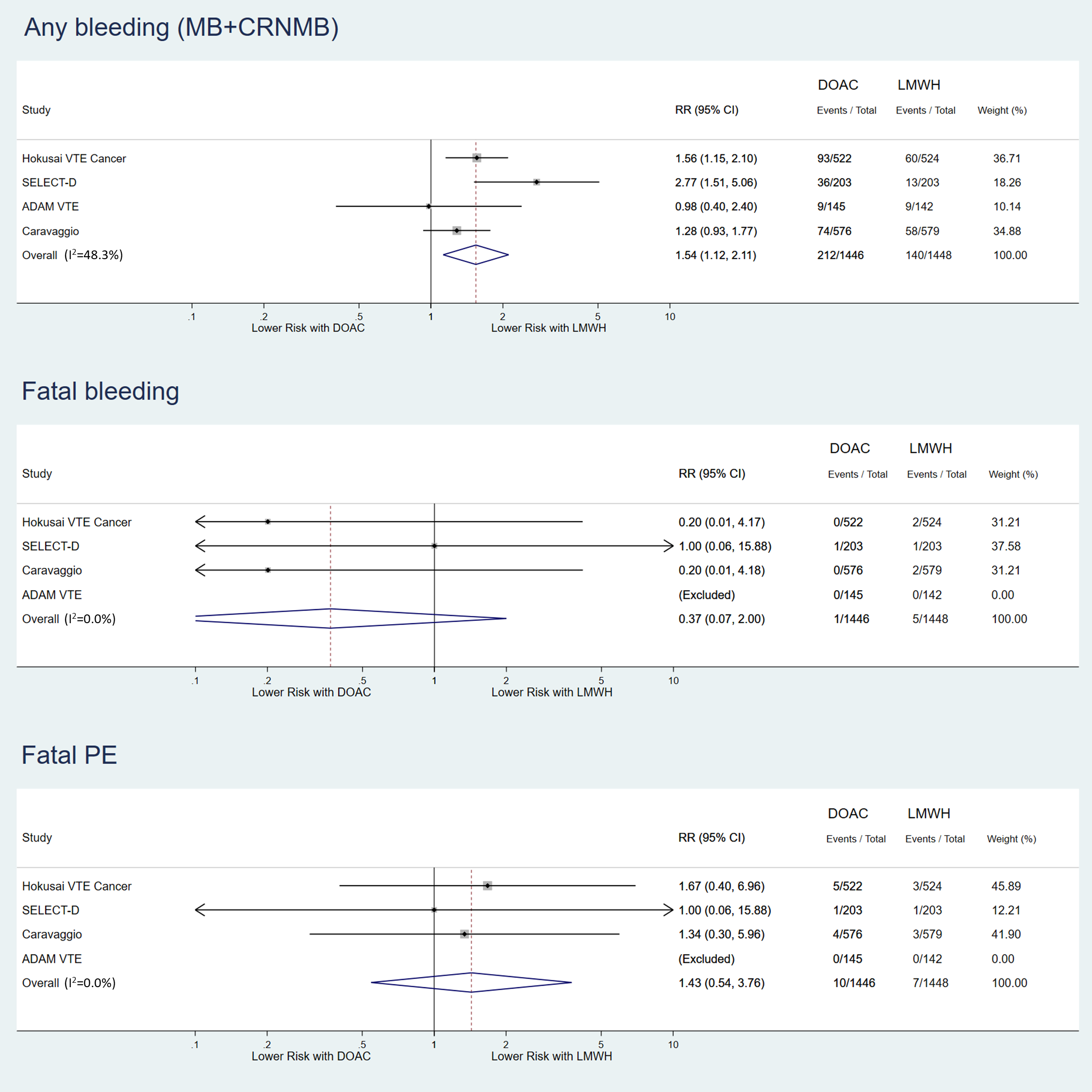

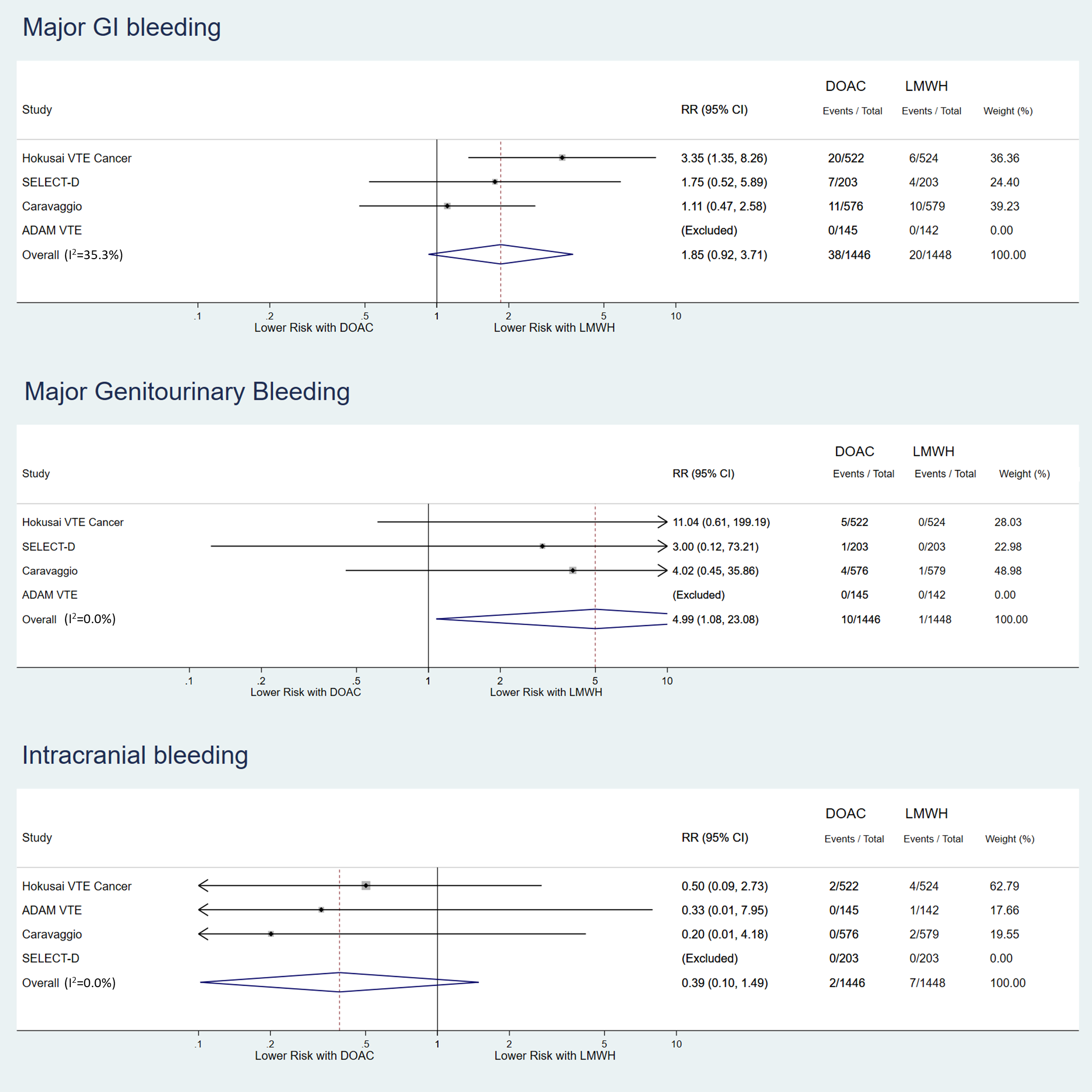


## Supplemental Figure S3: Sensitivity analysis including data from Hokusai VTE Cancer, SELECT-D and Caravaggio


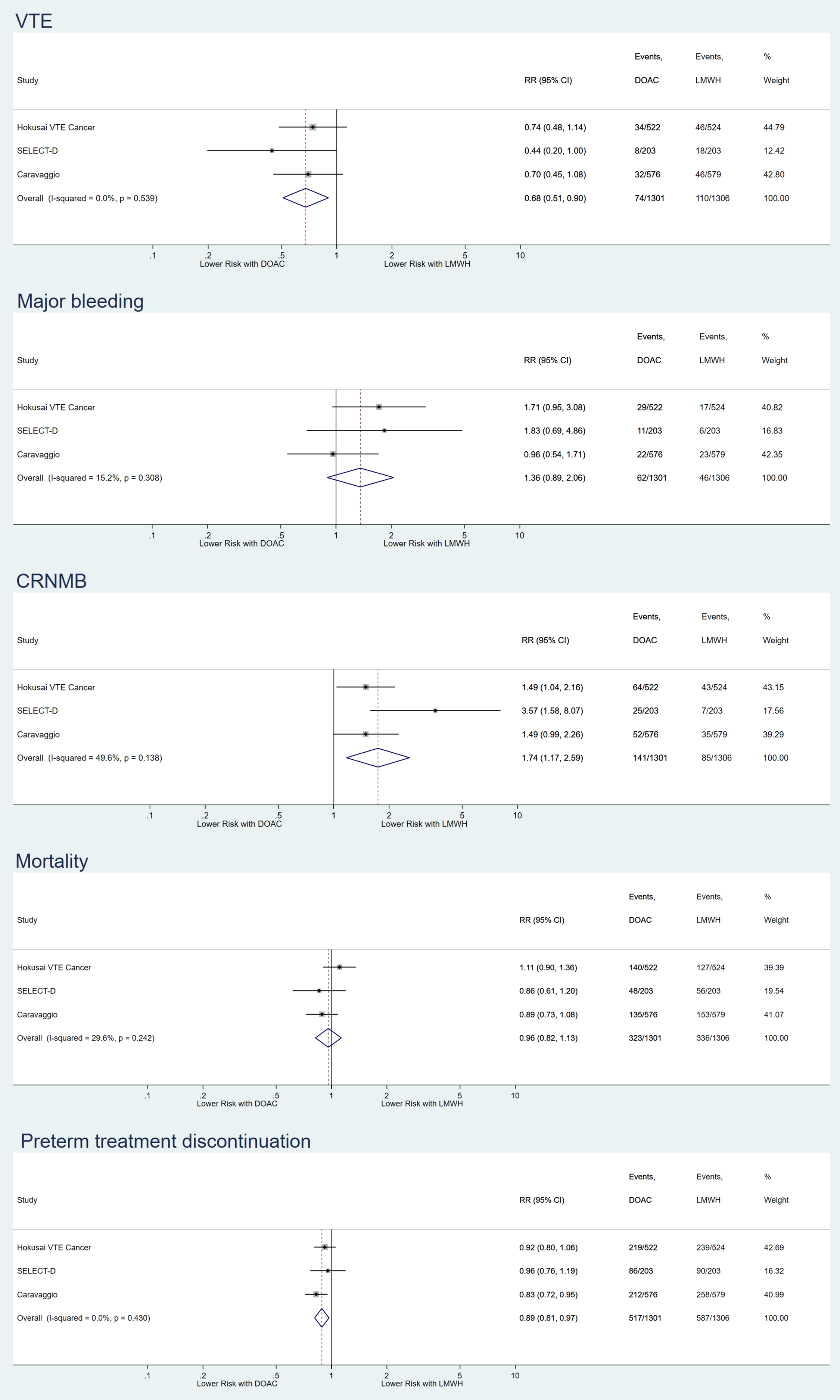


## Supplemental Figure S4: Sensitivity analysis for on-treatment period


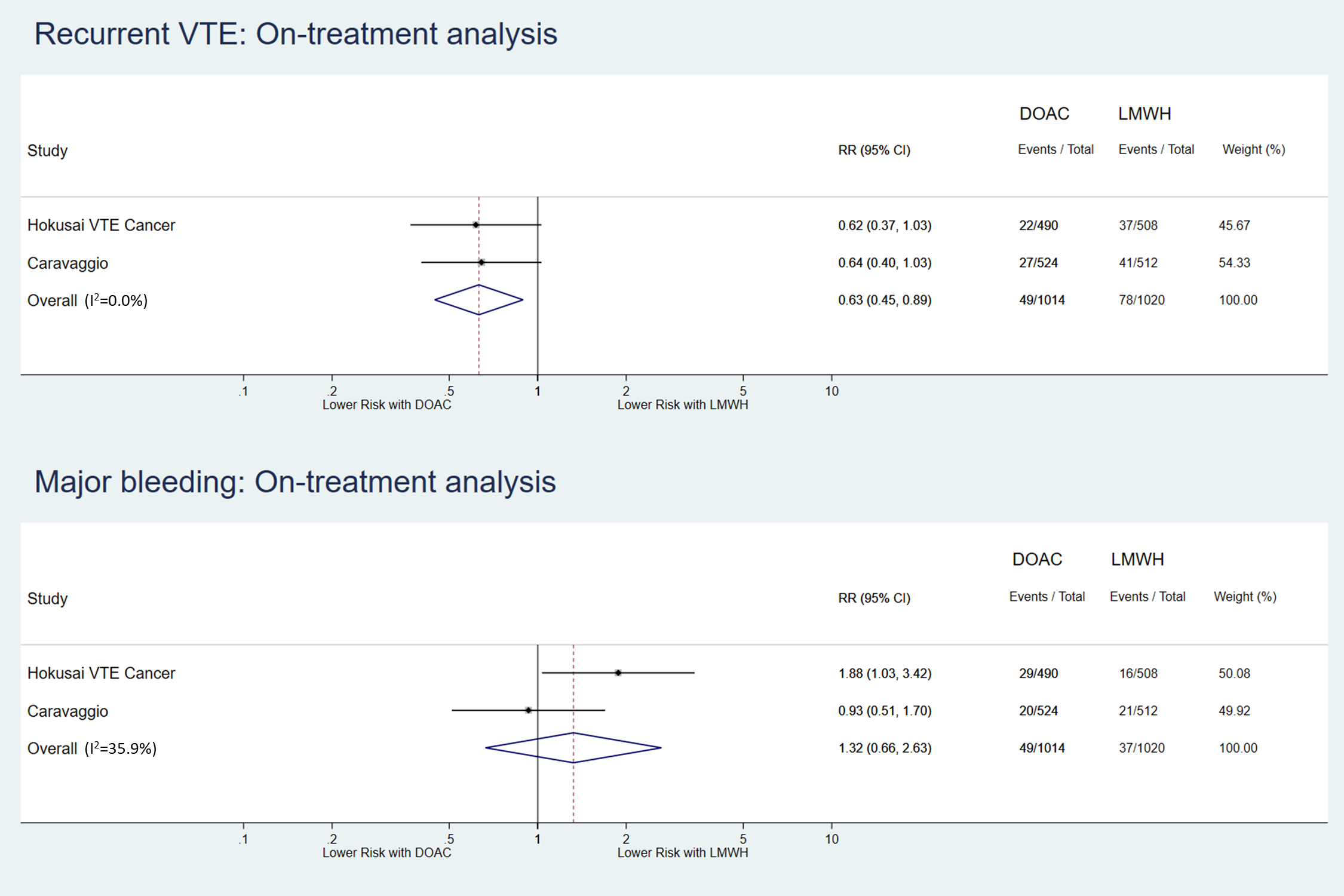

Supplement: Supplementary file 1 — Appendix S1 [file RTH2-4-550-s001.docx]
